# Interactions between HLA-G and HLA-E in Physiological and Pathological Conditions

**DOI:** 10.3389/fimmu.2014.00394

**Published:** 2014-08-22

**Authors:** Fabio Morandi, Vito Pistoia

**Affiliations:** ^1^Laboratory of Oncology, Istituto Giannina Gaslini, Genoa, Italy

**Keywords:** HLA-G, HLA-E, tumor, autoimmune disease, viral infections

## Abstract

HLA-G and HLA-E are immunoregulatory molecules that belong to HLA-Ib family. The role of these molecules in the control of the immune response has been extensively analyzed, both in physiological and pathological conditions. We have here summarized data present in the literature regarding the interaction of these molecules in different settings. These data suggested that HLA-G and -E co-operate in physiological conditions (i.e., establishment of an immune tolerance at maternal/fetal interface during pregnancy), whereas their role in the course of tumors or autoimmune/inflammatory diseases may be different or even opposite. Future studies aimed at investigating the interaction between HLA-G and HLA-E will help to clarify mechanism(s) underlying the regulation of immune effector cells in health and disease.

## Introduction

HLA-G and HLA-E belong to non-classical HLA-class Ib family that also includes HLA-F and HLA-H. In contrast with classical HLA-Ia molecules (HLA-A, -B, and -C), these molecules display a limited polymorphism, with a small number of proteins encoded by few alleles (http://hla.alleles.org/nomenclature/stats.html, data are summarized in Table S1 in Supplementary Material). Moreover, the function of HLA-class Ia and Ib molecules is different. In fact, HLA-class Ia molecules bind peptides generated from cytoplasmic proteins (in general represented by viral or tumor-associated antigens) and interact with antigen-specific T-cell receptor expressed on cytotoxic CD8^+^ T-cells, leading to the recognition of virus infected or transformed cells (1). In addition, HLA-class Ia molecules can interact with killer-inhibitory receptors expressed on NK cells, thus modulating NK cell functions (2).

HLA-class Ib molecules are also able to bind peptides generated from intracellular antigens, but the main function of these molecules is to modulate the immune response by interacting with specific inhibitory receptors expressed on different immune effector cells (3).

HLA-G is the best characterized among HLA-class Ib molecules. Seven different isoforms are encoded by the same primary mRNA through alternative splicing. Four isoforms (HLA-G1, -G2, -G3, and -G4) retain the transmembrane domain and therefore are membrane-bound, whereas the other three isoforms (HLA-G5, -G6, and -G7) retain the intron-4 and lose the transmembrane domain, and are therefore released as soluble molecules. In addition, soluble(s) HLA-G can be generated from membrane-bound molecules, through the cleavage operated by metalloproteases (4). In this respect, Rizzo et al. have recently reported that metalloprotease 2, but not 9, is involved in this process (5).

HLA-G expression is extremely restricted, being detected in physiological conditions in placental trophoblast cells at maternal–fetal interface during pregnancy (6), in thymus (7), cornea (8), nail matrix (9), pancreas (10), monocytes (11), erythroid (12), and endothelial precursors (13). However, HLA-G expression can be also detected in different immune cell populations, such as T-cells (14, 15), antigen-presenting cells (15–17), and in immunoregulatory cell populations, such as mesenchymal stem cells (18, 19). Nevertheless, HLA-G is up-regulated in different pathological conditions, such as transplantation, tumors, viral infections, and inflammatory diseases (20, 21).

The role of this molecule is to regulate the immune response, both in physiological and pathological conditions. This feature is important at maternal–fetal interface, to avoid the lysis of semiallogeneic fetal tissue by maternal NK cells (22–25). Similarly, in transplanted patients, an increased expression of surface HLA-G (26) and an augmented concentration of serum sHLA-G (27) may protect the transplanted organs from the rejection by the host’s immune system. Conversely, HLA-G expression on transformed cells (tumor cells and virus-infected cells) provides them with an immune escape mechanism, avoiding the recognition and lysis by cytotoxic immune effectors, such as NK cells and cytotoxic T lymphocytes (28).

The immunoregulatory properties of this molecule are related to the inhibition of the function of different immune cell populations, such as T- and B-lymphocytes, NK cells, and antigen-presenting cells. Such inhibition is mediated by the interaction of HLA-G molecules with at least four inhibitory receptors expressed on immune effector cells: immunoglobulin-like transcript (ILT)2 on NK cells, T- and B-lymphocytes; ILT4 on myeloid cells; KIR2DL4 on NK cells and T-lymphocytes; and CD160 on NK cells and T-lymphocytes (4).

The expression of HLA-E mRNA can be virtually detected in all nucleated cells. However, the surface expression of HLA-E, that requires the presence of peptides derived from other HLA-class I molecules and β2-microglobulin, is extremely restricted and it has been related to cell activation (29). In fact, the function of HLA-E is to bind peptides derived from the leader sequence of HLA-class I molecules (HLA-A, -B, -C, and -G) and to present them to NK cells through the interaction with the inhibitory receptor CD94/NKG2A, thus inhibiting NK cell lysis against cells that express normal levels of HLA-class I molecules. Conversely, cells with low levels of HLA-class I expression generate low levels of HLA-class I derived peptides and consequently display a low level of HLA-E, thus allowing NK cell lysis (30). HLA-E can also interact with CD94/NKG2C activating receptor on NK cells, in particular when it binds peptides generated from HLA-G. This feature is employed to activate NK cell lysis against HLA-G^+^ trophoblast cells during placental invasion, leading to tissue remodeling (31). However, it has been demonstrated that HLA-E affinity to the inhibitory NKG2A/CD94 receptor is sixfold higher than its affinity to the activating NKG2C/CD94 receptor (32). Finally, HLA-E can present different peptides to HLA-E restricted effector cells. Romagnani et al. have identified a CD8^+^ T-cell subset that recognized different peptides associated to HLA-E on allogeneic cells, thus highlighting their importance in transplantation and anti-tumor immune responses (33). Moreover, it has been reported that HLA-E present CMV-derived peptides to a subset of HLA-E restricted CMV-specific CD8 + T-cells (34). This feature may be relevant in the control of viral infections, since cytomegalovirus is able to avoid the control of conventional CTL or NK cells. On the other hand, Jiang et al. have demonstrated that peptides derived from the signal peptide of Hsp60 and loaded on HLA-E are recognized by a subset of CD8^+^ regulatory T-cells that are able to control self-reactive T-cells. The loss of this recognition may lead to the development of autoimmune diseases (35).

In this review, we summarize for the first time data present in literature regarding the interaction between HLA-G and HLA-E, focusing on the role of this interaction in the control of the immune response both in physiological and pathological conditions.

## HLA-G and HLA-E Co-Operate in Physiological Conditions

Several authors have demonstrated that HLA-G can influence and modulate HLA-E expression. In particular, the expression of different isoforms of HLA-G may affect surface HLA-E expression, which depends on the availability of peptides derived from HLA-G molecules and other HLA-class I molecules. In this view, it has been demonstrated that HLA-E surface expression was higher in cells transfected with HLA-G1 or -G3 than in untransfected cells. Moreover, HLA-E expression was higher in cells transfected with HLA-G1 than in cells transfected with HLA-G3 (36). Similarly, Ulbrecht et al. have demonstrated that the truncated isoforms of HLA-G (HLA-G2, -G3, and -G4) are less efficient to provide peptides to HLA-E molecules. Consequently, HLA-E expression is lower in cells that express high levels of HLA-G truncated isoforms than in cells expressing HLA-G1 (37). This effect was likely related to the ability of full-length transmembrane isoforms to act as chaperone for HLA-E molecules, since the leader sequence, that generates HLA-E binding peptides, is identical across different HLA-G isoforms. However, data obtained by Sala et al. are partially in contrast with this conclusion. They transfected JAR cell line with HLA-G*0105N allele, which encodes a truncated isoform containing the leader peptide, the complete α1 domain, and the first half of the α2 domain. Although this truncated HLA-G1 protein is rapidly degraded, its leader sequence after cleavage might still be available for binding to the HLA-E molecule. In fact, transfected cells do not express HLA-G1 molecule on the surface, but express a functional HLA-E molecule that is capable to inhibit NK cell lysis by interacting with CD94/NKG2A receptor (38).

HLA-G and HLA-E are physiologically co-expressed on different cell populations and can interact to modulate the immune response. In this regard, Ishitani et al. have demonstrated that HLA-E expression in trophoblast cells was strongly related to HLA-G expression. In fact, surface expression of HLA-G was found in extravillous trophoblasts, whereas sHLA-G production was found in all placental trophoblasts, including villous cytotrophoblasts and syncytiotrophoblasts. HLA-E expression was detected in all cells that expressed either form of HLA-G, suggesting that HLA-E requires peptides derived from all isoforms of HLA-G to be expressed (39). Similarly, Shaikly et al. have demonstrated that HLA-G and HLA-E co-localize on the surface of trophoectodermal cells, and may regulate implantation through the regulation of the effector functions of uterine leukocytes, by interacting with different receptors expressed by different cell populations, leading to an addictive effect (40). Moreover, it has been recently demonstrated that mesenchymal stromal cells derived from gestational tissue (in particular derived from the cord blood) are poorly immunogenic, and this feature is related to the co-expression of HLA-G and HLA-E on their cell surface (41). Similarly, induced pluripotent stem cells (iPSCs) express low levels of classical HLA-class I molecules, but express high levels of HLA-G and HLA-E and are able to avoid the recognition of HLA-restricted cytotoxic T-cells, which become anergic when co-cultured with iPSCs (42).

## HLA-G and HLA-E Interaction may be Relevant during Cancer and Viral Infections

HLA-G and HLA-E can co-operate to establish an immunosuppressive microenvironment in human tumors and viral infections, facilitating the escape of transformed cells from the recognition by the immune system.

In this view, de Kruijf et al. have demonstrated that in patients with breast cancer either HLA-G or HLA-E expression correlated with worse overall and event-free survival. This was observed only in patients with tumors that display a loss of classical HLA-I molecules, thus suggesting that it may occur only when activated NK cells are present. Notably, patients with tumors co-expressing HLA-G and -E display the worst clinical outcome, thus suggesting that the two molecules may co-operate shutting down NK cell-mediated anti-tumor immune response (43). Similarly, it has been demonstrated that HLA-G and -E co-expression correlated with the presence of metastasis and with a worse event-free and overall survival in patients with colon cancer, irrespective of the expression of HLA-class Ia molecules (44). Nevertheless, Malmberg et al. have demonstrated that short-term ovarian carcinoma cell lines treated with IFN-γ become resistant to CTL-mediated lysis. Such effect was mediated by increased HLA-G expression, which in turn leads to up-regulation of HLA-E on tumor cells. Surface HLA-E inhibits CTL activity by interacting with the inhibitory receptor CD94/NKG2A (45).

In contrast with these studies, several groups have demonstrated that HLA-G and -E may have different or even opposite roles in tumor progression. In this respect, da Silva et al. have demonstrated that HLA-G was overexpressed in the majority of biopsies derived from patients with breast cancer, whereas HLA-E expression was detected at low level in a small number of biopsies, thus suggesting that, at least in this cohort of breast cancer patients, HLA-G and -E interaction does not likely take place (46). Similarly, HLA-G is specifically expressed in renal cell carcinoma and not in normal renal parenchyma, whereas HLA-E is expressed in both normal and pathological tissues. Moreover, a better relapse-free survival was associated with a low HLA-G expression and with a high HLA-E expression, thus suggesting a divergent role of these molecules in the progression of this type of tumor (47). On the contrary, Silva et al. have demonstrated that, in patients with laryngeal lesions, HLA-G expression was detected in benign and premalignant lesions and not in invasive carcinomas, whereas HLA-E expression correlated with lesion grade, with a high expression in the draining lymph nodes of malignant lesions. Also, in this case, however, an opposite role of HLA-G and -E in tumor progression was demonstrated (48). Similarly, in patients with cervical carcinoma, HLA-G expression was detected in atypical glandular cells of undetermined significance and disappeared in cervical intraepithelial neoplasia (CIN) and invasive cancer, whereas HLA-E expression increased from CIN1 to CIN3 grade and the highest HLA-E expression was detected in invasive cancer, thus suggesting that HLA-E, rather than HLA-G, has a role in immune escape of transformed cells (49). Finally, HLA-G and HLA-E expression was detected in about 70% of biopsies from glioblastoma cells, and co-expression was detected in 36% of cases. A high HLA-E expression was related to a better overall survival, whereas no correlation was found between HLA-G expression and clinical outcome of patients (50).

HLA-G and -E can also co-operate in the tumor microenvironment to induce local anergy (51). It has been demonstrated that tumor-associated macrophages (TAM) express HLA-G on their surface (52). HLA-G expressed and/or released by TAM may interact with inhibitory receptors on NK cells stimulating the release of pro-angiogenic cytokines, as reported (53). Kren et al. have demonstrated that TAM may also express HLA-E (54), which interacting with the inhibitory receptor CD94/NKG2A on NK cells may further stimulate the release of immunosuppressive cytokines from NK cells (55). Thus, HLA-E may collaborate with HLA-G in the protection of TAM from NK cell lysis (30, 56) and in the establishment of a tolerogenic tumor microenvironment.

HLA-G and HLA-E interaction may also take place during viral infections. In this view, it has been demonstrated that rabies virus is able to up-regulate both HLA-G and -E expression in infected human neuronal precursors, and both molecules facilitate the immune escape of infected cells (57). Similarly, Vasireddi and Hilliard have demonstrated that, in contrast with other herpesviruses, herpes B virus does not downregulate the expression of HLA-Ia molecules. In contrast, HLA-G and -E expression is significantly up-regulated in infected cells, thus again suggesting a role of both molecules in the escape of infected cells from the recognition of the immune system (58).

## HLA-G and HLA-E may have Opposite Roles in Inflammatory/Autoimmune Diseases

Only few data are present in the literature regarding the role of both HLA-G and -E in inflammatory/autoimmune diseases. However, data obtained from our group in patients with juvenile idiopathic arthritis (JIA) and multiple sclerosis suggest that HLA-G and -E may have either an opposite or a synergistic role in the course of these pathological conditions.

In fact, we have demonstrated that in JIA patients, HLA-G may be more relevant as soluble molecule in the biological fluids, since serum levels of sHLA-G are decreased in patients as compared to controls. This may lead to an uncontrolled activation of immune effector cells, which eventually migrate to the synovium, causing tissue damage. In contrast, HLA-E appears to be more important as surface molecule, since its expression is higher on infiltrating synovial cells (mostly on B cells and monocytes) than in peripheral blood counterparts. This feature may be relevant to protect autoreactive cells from NK cell-mediated lysis, thus exacerbating local inflammation. Nevertheless, sHLA-E concentration in synovial fluid correlated with disease severity, thus suggesting that this molecule may represent a marker of cell activation (59).

In contrast with these observations, data obtained in multiple sclerosis patients suggested that HLA-G and HLA-E can co-operate in the resolution of inflammation. In fact, concentration of both sHLA-G and sHLA-E was higher in sera from patients than controls (represented by patients with other neurological disorders). More importantly, intrathecal synthesis of HLA-G and -E was detected, and concentration of both molecules was increased in cerebrospinal fluid (CSF) from MS patients as compared to controls. Moreover, sHLA-E concentration was higher in clinically stable patients than in those with clinically active disease. Finally, CSF samples inhibited *in vitro* NK- and CTL-mediated lysis. Such inhibition was higher using samples containing both HLA-G and -E than samples containing HLA-G or HLA-E, or devoid of both molecules. Taken together, these data suggested that HLA-G and HLA-E co-operate in the inhibition of immune effector cell function, and may have a role in the resolution of neuroinflammation (60).

## Concluding Remarks

**Figure 1:**
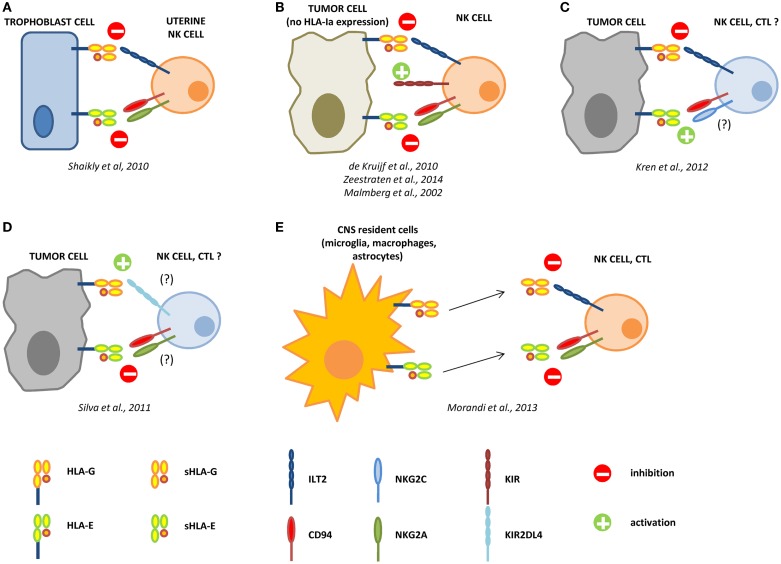
**Interactions between HLA-G and HLA-E in the control of the immune response**. During pregnancy, HLA-G and -E are both expressed by trophoblast cells and co-operate in the inhibition of NK cell functions, by interacting with ILT2 and CD94/NKG2A receptors, respectively **(A)**. In different tumors, the loss of HLA-class Ia molecules activate NK cells through KIR ligand mismatch. HLA-G and -E co-operate in the inhibition of activated NK cells in the tumor microenvironment, facilitating the escape of tumor cells from NK cell recognition **(B)**. In renal cell carcinoma, HLA-G expression correlates with worse prognosis, whereas HLA-E expression represents a favorable prognostic marker. We can speculate that in this case HLA-G preferentially interacts with inhibitory receptors on NK cells and CTL, whereas HLA-E possibly interacts with CD94/NKG2C activating receptor on immune effector cells **(C)**. On the contrary, in laryngeal carcinoma, HLA-G predicts a good prognosis, whereas HLA-E is associated with worse prognosis. In this case, we speculate that HLA-G may predominantly interact with KIR2DL4 activating receptor, whereas HLA-E interacts with CD94/NKG2A inhibitory receptor on NK cells and CTL **(D)**. In multiple sclerosis patients, HLA-G and HLA-E are expressed and released by resident cells in the central nervous system (CNS), and both soluble molecules co-operate in the inhibition of NK cells and CTL function, by interacting with inhibitory receptors **(E)**.

We have here summarized for the first time the interaction between HLA-G and -E in different settings (data are summarized in Figure 1). We can conclude that, in physiological conditions, HLA-E expression is strongly related to HLA-G, and normally both molecules are involved in inducing anergy of activated immune effector cells (mostly NK cells). Conversely, the interaction of these molecules in pathological conditions may be variable, ranging from a strong correlation and co-operation to an opposite function and role in the progression of the disease (see Table 1). Future studies aimed at a better knowledge of these interactions may explain the mechanisms underlying the establishment of an immunosuppressive microenvironment.

**Table 1:** **Summary of HLA-G and HLA-E interactions in pathological conditions**.

	Disease	Co-operation	Correlation	No correlation	Opposite role
Tumors	Breast cancer (43)	x			
	Colon cancer (44)	x			
	Ovarian carcinoma (45)	x			
	Breast cancer (46)			x	
	Renal cell carcinoma (47)				x
	Laryngeal carcinoma (48)				x
	Cervical carcinoma (49)			x	
	Glioblastoma (50)			x	
Viral infections	Rabies virus (57)		x		
	Herpes B virus (58)		x		
Autoimmune disease	Juvenile idiopathic arthritis (59)			x	
	Multiple sclerosis (60)	x			

## Conflict of Interest Statement

The authors declare that the research was conducted in the absence of any commercial or financial relationships that could be construed as a potential conflict of interest.

## Supplementary Material

The Supplementary Material for this article can be found online at http://www.frontiersin.org/Journal/10.3389/fimmu.2014.00394/abstract
