# Supplementary material for: Interactions between HLA-G and HLA-E in Physiological and Pathological Conditions
Source: Front Immunol. 2014 Aug 22;5:394. doi: 10.3389/fimmu.2014.00394 (PMC4141331; doi:10.3389/fimmu.2014.00394)
Supplement: Supplementary file 1 [file TableS1.PDF]

Table S1 | Alleles and proteins of HLA-class I molecules.

	HLA-class I molecules							
	HLA-class Ia			HLA-class Ib			Pseudogenes	TOT
	A	B	C	E	F	G	(H–X)	
Alleles	2884	3590	2375	15	22	50	40	8976
Proteins	2041	2668	1677	6	4	16	0	6412
Nulls	133	119	71	0	0	2	0	325
